# Facile Analytical Methods to Determine the Purity of Titanium Tetrachloride

**DOI:** 10.1155/2018/6470196

**Published:** 2018-08-19

**Authors:** Dongwook Lim, Sang-Deuk Kim, Hyungseok Kong, Daehyeon Nam, Sang Eun Shim, Sung-Hyeon Baeck

**Affiliations:** Department of Chemistry and Chemical Engineering, Center for Design and Applications of Molecular Catalysts, Inha University, Incheon 402-751, Republic of Korea

## Abstract

We propose a simple method to investigate both the qualitative and quantitative properties of titanium tetrachloride. The selection and concentration of the employed solvent were found to be very important in the analysis of highly reactive titanium tetrachloride (TiCl_4_). Herein, we employed various concentrations of an acid solution to serve as a stabilizing medium. Qualitative analysis was performed via Fourier transform-infrared spectroscopy (FT-IR) and scanning electron microscope-energy dispersive spectroscopy (SEM-EDS). Additionally, the quantitative analysis was performed via inductively coupled plasma optical emission spectroscopy (ICP-OES). We concluded that both the qualitative and quantitative properties of titanium tetrachloride could be easily measured using a specific acidic solvent as a medium.

## 1. Introduction

Various efforts and attempts to reduce energy consumption have been applied to fields of industry and research. Endeavours with regard to improving energy efficiency within the transportation industry have attracted considerable attention toward materials. In this respect, titanium and titanium complexes have been recognized as being appropriate materials, and the consumption of titanium has been soaring across the world [1]. Pure titanium and titanium complexes have been widely used as raw materials for aerospace fuselages, airplane fuselages, and aero-engine compressor disks (requiring high strength, light weight) for high-temperature stability and corrosion resistance to enhance energy consumption efficiencies [2–6].

Besides the fields mentioned above, titanium complexes, especially titanium dioxide, are considered to be versatile metal oxides due to their wide range of applications [7–13], and high purity titanium tetrachloride is strongly required for the synthesis of titanium dioxide. In particular, size, purity, and phases of titanium dioxide are key factors affecting key properties. Various methods have been used with regard to producing ultrafine crystalline titanium dioxide, such as the sol-gel method [14, 15], sputtering [16], homogeneous precipitation in an acidic solution [17], heat treatment of aqueous titanium tetrachloride (TiOCl_2_) [18], hydrolysis of titanium tetrachloride [19, 20], and the oxidation of titanium tetrachloride at high temperature (CVD) [21, 22]. The above-mentioned ways to prepare titanium dioxide generally use titanium tetrachloride as a raw material. However, it is well known that quantitative and qualitative analyses to determine the purity of TiCl_4_ are very difficult.

Titanium tetrachloride is an inorganic liquid transition metal halide compound at room temperature. The colour of pure titanium tetrachloride (99.9%) is transparent, but that of crude titanium tetrachloride is light yellow. Noteworthy roles of titanium tetrachloride involve its use as a raw material for the production of pure titanium metal and titanium dioxide as mentioned above [4–6, 14, 17–22]. Naturally, the purity of titanium tetrachloride is a significant factor toward achieving high purity titanium dioxide and titanium metal. However, titanium tetrachloride is an extremely reactive compound, releasing highly corrosive gases such as hydrogen chloride (HCl) whenever the material is exposed to water or moisture in the atmosphere [23, 24]. For these reasons, it is difficult to analyse the purity of titanium tetrachloride. The purpose of this study was to optimize a purity analysis method of the highly reactive titanium tetrachloride material.

We proposed a facile analytical method to determine the purity of titanium tetrachloride. Through a stabilization of titanium tetrachloride in various concentrated acidic chloride solutions, the stabilized samples were investigated via Fourier transform-infrared spectrometry (FT-IR), energy dispersive spectroscopy (SEM-EDS) for a qualitative analysis, and inductively coupled plasma optical emission spectrometry (ICP-OES) for the quantitative analysis. With a variety of commercial titanium tetrachloride samples, impurities in titanium tetrachloride were confirmed via qualitative analysis, and purities were established via quantitative analysis.

## 2. Materials and Methods

### 2.1. Reagents and Apparatus

High purity titanium tetrachloride was supplied by Junsei Chemical Co., Yakuri Pure Chemicals Co., Wako Chemical Co., Aldrich chemical Co., and Fluka. All titanium tetrachloride reagents were of analytical reagent grade (99.9%). The hydrochloric acid was purchased by Samchun Chemical Co. The used hydrochloric acid solution was of analytical reagent grade (35 wt. % HCl). To prepare the diluent for titanium tetrachloride, various concentrated hydrochloric acid (HCl) solvent solutions were prepared by diluting the stock acid solution with DI water. A 35 wt. % HCl solution was diluted with various quantities of DI water, and then 100 mL of 1, 3, 5, 7, 9, and 11 M diluted hydrochloric acid solutions was prepared.

Fourier transform-infrared vacuum spectrometer (FT-IR, A Bruker VERTEX 80V) was used to analyse the impurities in titanium tetrachloride. Golden gate single reflection diamond attenuated total reflectance (ATR) was used to examine the liquid-state titanium tetrachloride samples. A Hitachi S-4300SE field emission-scanning electron microscope (FE-SEM), especially with energy dispersive spectroscopy (EDS), was used to analyse the solid-state samples. A PerkinElmer Optima 7300DV inductively coupled plasma optical emission spectrometer (ICP-OES) was used to investigate the quantities of impurities in titanium tetrachloride.

### 2.2. Qualitative Analysis of Titanium Tetrachloride

**Figure 1:**
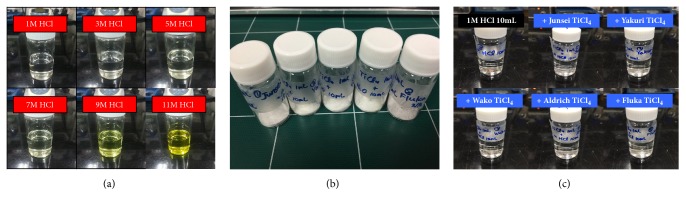
Stabilized titanium tetrachloride samples; (a) in various concentrated hydrochloric acid solutions, (b) hydrolyzed and calcined titanium tetrachloride (titanium oxide), and (c) titanium chloride samples and diluents prepared for ICP-OES analysis.

Liquid-state samples of stabilized titanium tetrachloride were prepared by diluting 1 ml of titanium tetrachloride with 10 mL of various concentrated hydrochloric acid solutions (1, 3, 5, 7, 9, and 11 M). All samples were stabilized and did not yield precipitates after 3 hours. The stabilized titanium tetrachloride samples in hydrochloric acid solution can be seen in Figure 1(a). To investigate the impurities in titanium tetrachloride via SEM-EDS, the materials were hydrolyzed in water. 1 mL of titanium tetrachloride was hydrolyzed with 10 mL of water. After the hydrolysis reaction, precipitation occurred and a white-coloured powder was synthesized at the bottom of the sample. The sample was dried at 80°C and calcined for 2 hours in an electric muffle furnace at 700°C. The hydrolyzed titanium tetrachloride (titanium oxide) samples can be seen in Figure 1(b).

### 2.3. Quantitative Analysis of Titanium Tetrachloride

A quantitative analysis of the impurities in titanium tetrachloride was carried out by ICP-OES. In order to analyse the quantities of impurities, 1 mL of titanium tetrachloride was diluted to 10 mL of a 1 M hydrochloric acid solution. The various titanium chloride samples diluted with 1 M hydrochloric acid can be seen in Figure 1(c).

## 3. Results and Discussion

### 3.1. Qualitative Analysis of Titanium Tetrachloride

Due to the explosive reactivity of titanium tetrachloride with water or the moisture in air, the material should be stabilized and diluted [23]. The liquid phase hydrolysis reaction of titanium tetrachloride can be described as follows:

(1) $$\begin{array}{c} TiCl_{4}+2H_{2}O\to TiO_{2}+4HCl \end{array}$$

**Figure 2:**
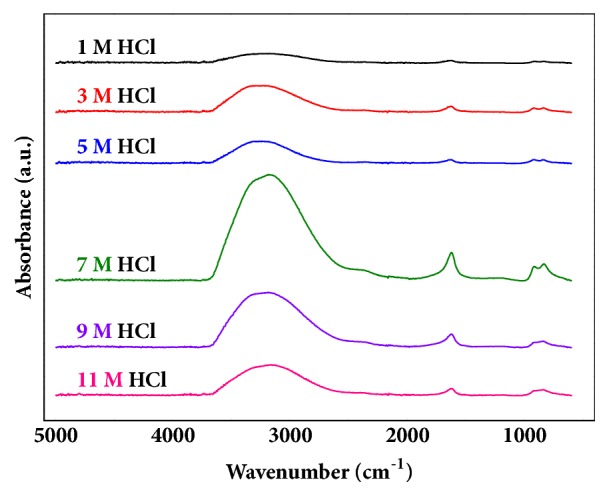
The effect of various hydrochloric acid concentrations for the qualitative analysis of titanium tetrachloride.

**Figure 3:**
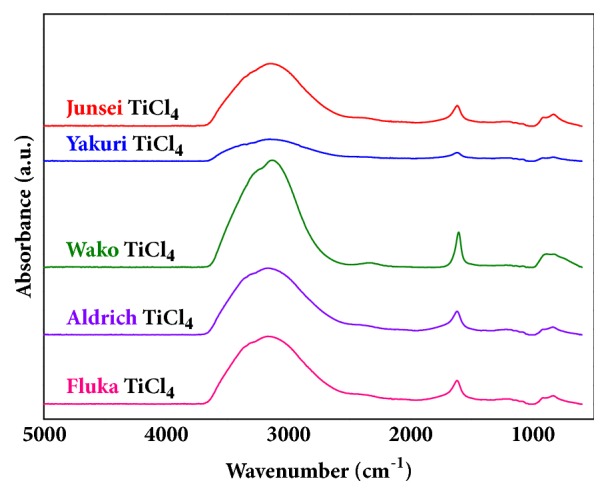
FT-IR analysis of various commercial titanium tetrachloride samples via dilution in 7 M hydrochloric acid.

A solution of hydrochloric acid is a proper stabilizer and diluent for the analysis, and the stabilization of titanium tetrachloride was performed by diluting the liquid with various concentrations of hydrochloric acid.  Figure 2 shows the FT-IR spectra of the diluted solutions, and the effects of various hydrochloric acid concentrations were clearly observed. The FT-IR spectrum intensities demonstrated that a 7 M hydrochloric acid solution was the proper diluent to prepare liquid-state samples for a qualitative analysis via FT-IR spectroscopy, maximizing the peak intensities. When titanium tetrachloride was diluted with hydrochloric acid, the titanium atoms in titanium tetrachloride were ionized and existed in the form of tetravalent titanium ions [25]. Lee et al. reported that changes in pH values for aqueous titanium tetrachloride induced a variety of tetravalent titanium ion concentrations [18]. Hydroxyl ions had a large effect on the pH values and reacted with the tetravalent titanium ions, yielding precipitated titanium dioxide. Therefore, it is important to achieve acidic conditions with regard to inhibiting precipitation and maintaining the stability of titanium ions [18]. However, as the concentration of hydrochloric acid increased up to 8 M or more, the transparent aqueous titanium tetrachloride solution changed to a white-coloured opaque solution without precipitation [18]. A 7 M concentration of hydrochloric acid solution was optimum to preparing stabilized samples for a qualitative analysis. These results could be applied toward the analysis of various commercial titanium tetrachloride samples, as shown in Figure 3. There were slight discrepancies in intensities, though all the band regions exhibited the same tendencies.

As shown in Figure 3, bands in the region of 3600-2500 cm^−1^ for the spectra of diluted titanium tetrachloride in aqueous solution were due to Ti-OH and -OH vibrations [26]. The band at 1642 cm^−1^ corresponded to H-O-H and Ti-H_2_O [27]. The IR spectrum around 820 cm^−1^ was observed to correspond to the TiOCl_2_ band as a by-product of the hydrolysis of titanium tetrachloride and water [17, 28, 29]. However, it is well known that other elements (Fe, Si, V, Sb, Mn, and Cr) exist as impurities in titanium tetrachloride. Due to the low concentrations of impurities in titanium tetrachloride, the impurities could not be analysed via FT-IR spectroscopy.

To further investigate the impurities in titanium tetrachloride, SEM-EDS analysis was employed to perform a qualitative analysis as shown in Table 1. Following the hydrolysis and calcination of titanium tetrachloride, a white-coloured titanium dioxide powder was prepared for EDS. While impurities such as Fe, Al, Si, Sb, Cr, and Mn were detected in titanium tetrachloride, other general impurities such as V, P, As, Ni, Cu, and Sn were not found.

### 3.2. Quantitative Analysis of Titanium Tetrachloride

From the qualitative analysis results, various impurities in titanium tetrachloride were determined. Quantitative analysis was performed to determine the quantity of impurities in titanium tetrachloride via ICP-OES as shown in Table 2. The experiments were carried out three times, and the standard errors were less than 3%. All data in Tables 2 and 3 represent mean values of three analyses. As mentioned in the experimental section, the titanium tetrachloride samples were prepared via dilution in 1 M hydrochloric acid. The results of the quantitative analysis indicated only cationic weights of impurities, which were composed of metal halides or metalloid halides. For these reasons, accurate weights of the impurities should be calculated with the addition of elemental chlorine. For instance, Fe existed in the form of FeCl_3_ within titanium tetrachloride. The atomic weight of Fe was 0.11*∗*10^−6^ g and that of 3Cl^−^ was 0.21*∗*10^−6^ g. Thereby, 0.32*∗*10^−6^ g of FeCl_3_ existed in titanium tetrachloride. Undergoing this process, the purity of titanium tetrachloride was determined by subtracting the mass of all the impurities within the prepared sample. As shown in Table 3, the purity values of titanium tetrachloride supplied by Junsei Chemical Co., Yakuri, Wako, Sigma Aldrich, and Fluka were 99.92, 99.93, 99.92, 99.91, and 99.92%, respectively. The results were consistent with the purity values reported by the titanium tetrachloride manufacturers.

## 4. Conclusions

Titanium tetrachloride samples for qualitative analysis were diluted with 7 M hydrochloric acid due to imparting the highest stability and yielding the highest intensity of FT-IR. Few impurities were detected, while the following impurities were not detected at any level: Fe, Si, V, Sb, Cr, and Mn. Another qualitative analysis (EDS) was utilized to determine the impurities through the hydrolysis of titanium tetrachloride. The results revealed that impurities such as Fe, Al, Si, Sb, Cr, and Mn existed in titanium tetrachloride. A quantitative analysis was also performed to know the concentrations of impurities by the qualitative analysis (ICP-OES). The analysis results indicated that the impurities in titanium tetrachloride existed at ppm concentrations. According to the results, the purity of titanium tetrachloride was almost 99.91~99.93%. This study established a facile analytical method to determine the purity of titanium tetrachloride via diluting and stabilizing with hydrochloric acid.

## Figures and Tables

**Table 1:** Atomic determination of impurities in titanium tetrachloride via SEM-EDS.

	Fe	Al	Si	Sb	Cr	Mn	V	P	As	Ni	Cu	Sn
Detection of element	O	O	O	O	O	O	X	X	X	X	X	X

**Table 2:** Quantitative determination of impurities in titanium tetrachloride via ICP-OES.

	Fe	Al	Si	Sb	Cr	Mn	V	P	As	Ni	Cu	Sn
ppm	0.11	0.52	3.99	41.3	1.86	0.05	-	-	-	-	-	-

**Table 3:** Purities of commercial titanium tetrachlorides.

Company	Junsei	Yakuri	Wako	Aldrich	Fluka
Purity (%)	99.92	99.93	99.92	99.91	99.92

## Data Availability

The data that support the findings of this study are available from the corresponding author upon reasonable request.
